# Combination of through-the-scope suturing and over-the-scope clips for closure of persistent gastrocutaneous fistula after gastrostomy tube removal (“X-lock technique”)

**DOI:** 10.1016/j.vgie.2025.05.004

**Published:** 2025-05-31

**Authors:** Sagar Shah, Alireza Sedarat, Adarsh Thaker

**Affiliations:** Vatche and Tamar Manoukian Division of Digestive Diseases, Department of Medicine, David Geffen School of Medicine at University of California Los Angeles, Los Angeles, California, USA

## Abstract

**Background and Aims:**

Persistent gastrocutaneous fistula (GCF) can occur after removal of gastrostomy tubes. Although surgical closure has historically been considered the most definitive treatment option, novel endoscopic devices have allowed for less-invasive closure techniques.

**Methods:**

We present 2 cases in which closure of persistent GCF was performed using a combination of through-the-scope suturing and an over-the-scope clip (OTSC). In the first case, the suturing system and OTSC are used simultaneously, whereas in the second case, they are used sequentially.

**Results:**

There were no immediate postprocedural adverse events after both procedures. Both patients resumed oral intake the day after the procedure was performed. The first patient has had no leakage from previous GCF for 2 months, and the second patient has not had recurrent leakage for over 12 months since closure.

**Conclusions:**

Combination of through-the-scope suturing and OTSC placement is a viable treatment option for chronic GCF after gastrostomy tube removal.

## Background and aims

Although removal of gastrostomy tubes (G-tubes) typically is straightforward and safe, a persistent gastrocutaneous fistula (GCF) can form in a minority of cases. In a study of adult patients with head and neck cancer who had G-tubes removed, 5.7% of patients developed persistent fistulas.^1^ The duration of G-tube placement has consistently been associated with risk of persistent GCF.^1^, ^2^, ^3^

Although surgical closure has historically been considered the most definitive treatment option, the emergence of novel endoscopic devices has led to noninvasive options for managing this adverse event. We hypothesized that recurrence of leakage after endoscopic closure could be related to difficulty obliterating the entire fistula opening using either an over-the-scope clip (OTSC) or suturing system alone. Recent data suggest that use of through-the-scope (TTS) suturing systems for repair of fistulas and perforations achieved clinical success rates ranging from 28% to 66%.^4^ Similar rates of persistent leakage have been described with use of an OTSC for defect closure.^5^ We present 2 cases in which, to address this potential limitation, closure of persistent GCF was performed using a combination of TTS suturing and an OTSC.

## Methods

### Case 1

A 69-year-old woman with a history of class III obesity and chronic hypoxic respiratory failure initially had a G-tube placed to facilitate enteral nutrition and medication administration while mechanically ventilated. The G-tube had been in place for more than 2 years. On the basis of discussions regarding goals of care with the patient and her family during an admission for aspiration, food intake by mouth was resumed, and the G-tube was removed to allow closure of the tract. The gastrostomy tract failed to close over the course of several months and a persistent GCF formed, leading to bothersome leakage. Given negative effects on the patient's quality of life, endoscopic closure was attempted.

### Case 2

A 32-year-old woman with a history of Smith-Lemli-Opitz syndrome with global developmental delay and dependence on tracheostomy and G-tube was admitted for issues with enteral feeding. Before admission, the patient was having unspecified issues with an old G-tube site. This G-tube was removed, and a new G-tube was placed at a different site. When the patient was feeding through the new tube, tube feed would come out through the old site. Despite use of total parenteral nutrition for more than a month, leakage continued through the GCF. Endoscopic closure was attempted using a combination of argon plasma coagulation to induce granulation and closure with an OTSC; however, leakage persisted and repeat closure was attempted.

### Procedural techniques

**Figure 1:**
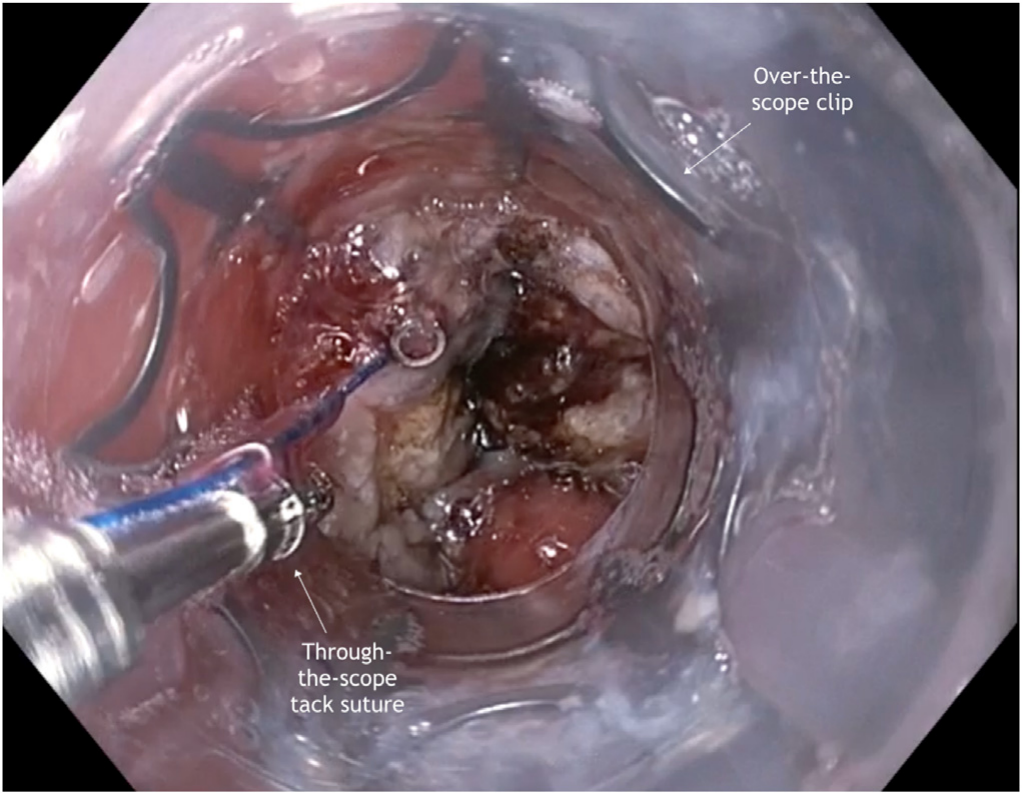
Use of through-the-scope tack suturing device within over-the-scope clip cap.

**Figure 2:**
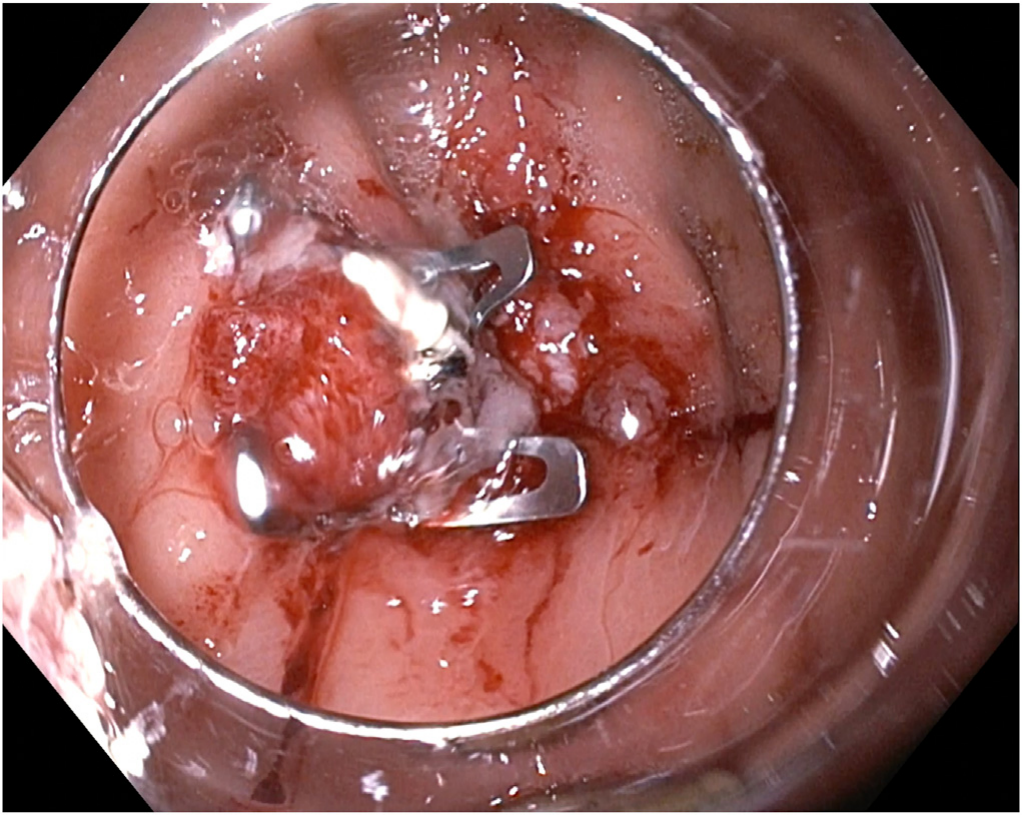
Appearance of fistula site after endoscopic suturing and placement of over-the-scope clip.

A standard gastroscope was passed into the gastric body where the internal opening to the GCF was visualized (Video 1, available online at www.videogie.org). To disrupt the epithelial layer of the fistula and promote granulation, the tract opening and surrounding mucosa were ablated using a 7F straight-fire argon plasma coagulation catheter with pulsed and forced coagulation. This was followed by use of a TTS-helical tack suturing system (X-Tack; Boston Scientific, Marlborough, Mass, USA) to reduce the size of the target for subsequent OTSC deployment. In the first case, an OTSC, which uses a beside-the-scope deployment cable (Padlock clip, Steris, Mentor, Ohio, USA) was used, so the TTS-suturing system and the OTSC were installed on the endoscope simultaneously (Fig. 1). The helical tack was driven into the mucosa roughly 5 mm from the edge of the fistula site before deployment. This process was repeated in purse-string fashion with 4 additional tacks. After placement, simultaneous tension to the suture and suction was applied to draw the apposed tissue into the OTSC. The OTSC was then deployed and suture cinch placed. We have internally described this combination as the “X-lock technique.” In the second case, an OTSC that uses the working channel for the deployment string (Ovesco clip; Ovesco AG, Tübingen, Germany) was used. This 12/6 gc clip required use of a therapeutic gastroscope. Therefore, the TTS-suture was completely deployed and cinched, followed by mounting, passage, and deployment of the OTSC over the smaller target (Fig. 2).

## Results

### Outcomes

There were no immediate postprocedural adverse events after either procedure. Both patients resumed oral intake the day after the procedure was performed. The first patient has had no leakage from before GCF for 2 months, and the second patient has not had recurrent leakage for 5 months since closure.

## Conclusions

These cases demonstrate successful combination of TTS suturing and OTSC placement for the treatment of chronic GCF after G-tube removal. One limitation of an OTSC is that it can be difficult to capture all the edges of a target defect, particularly those with larger diameters or irregular shapes. The TTS helical tacking system facilitates an initial tissue apposition device to reduce the target size for a subsequent OTSC, which may provide a more robust grasping force. Additional advantages of this combination therapy include the ability to perform the procedure with a standard gastroscope, its cost-effectiveness compared with over-the-scope suturing systems, and a favorable learning curve compared with traditional suturing. There are limited data comparing the 2 OTSC systems used in the video. The first system does not occupy the working channel, leaving it free for simultaneous TTS suture use, and the second system offers a larger variety of shapes and sizes. Although each system has its advantages, their effects on procedure ease, efficiency, technical success, and clinical success remain unknown.

## PATIENT CONSENT

The families of both patients included in this report were contacted to obtain permission to use de-identified endoscopic footage and clinical information for the purposes of a scientific publication.

## Disclosure

All authors disclosed no financial relationships.

## Supplementary data

Video 1 Endoscopic closure of chronic gastrocutaneous fistulas using a combination of through-the-scope suturing and over-the-scope-clips.
